# Efficacy of Tunnel Technique (TUN) versus Coronally Advanced Flap (CAF) in the Management of Multiple Gingival Recession Defects: A Meta-Analysis

**DOI:** 10.1155/2023/8671484

**Published:** 2023-04-06

**Authors:** Frank Mayta-Tovalino, Joshuan J. Barboza, Vinay Pasupuleti, Adrian V. Hernandez

**Affiliations:** ^1^Unidad de Revisiones Sistemáticas y Meta-Análisis (URSIGET), Vicerrectorado de Investigación, Universidad San Ignacio de Loyola, Lima, Peru; ^2^Vicerrectorado de Investigación, Universidad Norbert Wiener, Lima, Peru; ^3^Oxford PharmaGenesis, Inc., Newtown, PA, USA; ^4^Health Outcomes, Policy, and Evidence Synthesis (HOPES) Group, University of Connecticut School of Pharmacy, Storrs, CT, USA

## Abstract

**Objective:**

We systematically assessed the efficacy of tunnel technique (TUN) vs. coronally advanced flap (CAF) in the management of multiple gingival recession defects in adults.

**Methods:**

Five databases were searched until September 2021 for randomized controlled trials (RCTs) assessing TUN vs. CAF; grafts of interest were acellular dermal matrix (ADM) and connective tissue graft (CTG). Primary outcomes were root coverage (RC) and complete root coverage (CRC). Secondary outcomes were clinical attachment level (CAL), keratinized tissue width (KTW), probing depth (PD), and recession coverage (REC). Effect measures were risk ratio (RR) or mean difference (MD) with their confidence intervals (95% CI). Inverse variance methods and random-effects model meta-analyses were used. Subgroup analyses by the type of graft were performed. Quality of evidence was assessed using GRADE methodology.

**Results:**

Five RCTs (*n* = 173) were included, with a follow-up of 6 months for all outcomes. In comparison to CAF, TUN did not significantly reduce CRC (RR 0.65; 95% CI 0.002–176.7; *p* = 0.51) and did not increase RC (MD 0.99%; 95% CI −6.7 to 8.6; *p* = 0.80). In comparison to CAF, TUN showed no significant reduction of secondary outcomes. Subgroup analyses by type of graft showed no differences in comparison to primary analyses for primary and secondary outcomes. Three RCTs had a high risk of bias, and five RCTs had very low quality of evidence for all outcomes.

**Conclusions:**

In adults with gingival recessions, TUN had similar primary and secondary outcomes in comparison with CAF. Subgroup analyses by the type of graft did not affect main conclusions. More RCTs with better design are needed to further characterize the effects of TUN vs. CAF in the treatment of multiple gingival recession defects.

## 1. Introduction

Gingival recessions (GRs) are atrophic periodontal changes, and about 6 out of 10 young adults develop them [1]. These GRs show root surfaces partially or completely without evidence of an active inflammatory process [2]. Some of their risk factors are smoking, oral piercings, gingival inflammation, and frequent tooth brushing [3]. Although GRs usually generate an esthetic problem, they have been associated with dentine hypersensitivity, caries, cervical wear, and accumulation of dental plaque [3]. A study estimated that 58% of US adults have GRs <1 mm in male and the elderly [4]; however, in South American countries such as Brazil and Peru, GRs are even more frequent: 83% and 73% of adults, respectively [5].

Coronally advanced flap (CAF) is a traditional surgical procedure designed to achieve complete root coverage (RC) on single or multiple, continuous, or adjacent GRs [6]. This technique consists of two oblique incisions, begins from the distal and medial sides of the compromised teeth, and is projected to the alveolar mucosa. The flap has a split-thickness approach which is made to respect gingival and hard tissue [7]. However, another GR treatment is the newest tunnel technique (TUN), which is a minimally invasive procedure with no requirement of performing any vertical releasing incisions and leaves the interdental papillae intact [8]. TUN is designed to treat multiple and large GR that are usually found in the jaws where RC is difficult to obtain. In addition, TUN helps to maintain an adequate and constant blood irrigation in order to ensure an excellent adaptation of the graft in the receiving area [9].

Both RC techniques can use different types of grafts. One of the most used is connective tissue graft (CTG), which is considered as a gold standard for increasing keratinized soft tissue gums; its main disadvantage is that it requires a donor area and may have postsurgical complications [10]. Another type of graft is acellular dermal matrix (ADM), a specific type of CTG that is obtained through a decellularization mechanism to preserve the extracellular matrix. Generally, this type of graft serves as a scaffold for cells to proliferate and thus favors postsurgical revascularization [11–18].

For instance, a previous meta-analysis performed by Tavelli et al. [12] evaluated the efficacy of TUN compared to CAF in randomized controlled trials (RCTs). The authors included six RCTs in their meta-analysis and concluded that CAF showed superior outcomes such as complete RC and keratinized tissue width in comparison to TUN when the same graft (CTG or ADM) was used.

We systematically assessed the efficacy of TUN vs. CAF with two different grafts (ADM or CTG) in the treatment of multiple GR defects.

## 2. Materials and Methods

The protocol of the systematic review has been previously submitted in PROSPERO (CRD42019145355). We reported our study in accordance with the PRISMA (Preferred Reporting Items for Systematic reviews and Meta-Analysis) guidelines [13].

### 2.1. Search of Studies

We searched in Web of Science, Medline-Ovid, PubMed, Scopus, and Embase until September 18, 2021. There were no language restrictions. The search strategy was adapted for each database and are available in Supplementry Materials.

### 2.2. Eligibility Criteria

We selected RCTs evaluating adults with multiple GRs of Miller Class I, II, and III and assessed the comparison of TUN vs. CAF for RC on outcomes at 3, 6, and 12 months after baseline. On the other hand, grafts of interest were ADM or CTG. Besides, cohort studies, case reports, narrative reviews, and meta-analysis were excluded.

### 2.3. Outcomes

Primary outcomes were complete root coverage (CRC, dichotomous, defined as gingiva position at the cervical level of the teeth measured as yes/no), and root coverage (RC, continuous, measured in mean of % of the RC after the clinical procedures). Secondary outcomes were clinical attachment level (CAL, distance from the *cement–enamel* junction (CEJ) to the gingival margin (GM), measured in mm), keratinized tissue width (KTW) (measured in mm of dimension of thickness of the keratinized gingiva), probing depth (PD) (measured in millimeters of the dimension of the depth in the moment of the periodontal evaluation with a periodontal probe), and recession coverage (REC) (measured in millimeters of the dimension of the REC using periodontal probe). Author definitions described in each RCTs were used.

### 2.4. Selection of Studies

Two authors (JJB, FMT) independently assessed available records according to the inclusion and exclusion criteria and selected by the title, keywords, and abstract of reports identified through electronic searching. Then, full-text articles were evaluated. Remaining discrepancies were discussed with the fourth author (AVH).

### 2.5. Data Extraction and Management

Data were independently extracted by two authors (JJB, FMT). We used an extraction format designed according to the data and characteristics related to the included studies. All discrepancies were resolved by consensus with the fourth author (AVH). We decided not to include in the analysis data from studies in which the information was incomplete, and we contacted the corresponding study authors to provide appropriate clarification. We extracted per study the following variables: first author, year, trial phase, country, number of participants overall and per intervention arm, type of intervention and control and relevant details, and primary and secondary outcomes per intervention arm.

### 2.6. Risk of Bias Assessment

The 2019 Cochrane risk of bias (RoB) tool 2.0 tool was used to assess RoB per RCT [14]. This tool evaluates five domains of bias: randomization process, deviations from intended interventions, missing outcome data, measurement of the outcome, and selection of the reported result. Each domain and each RCT were rated as having low RoB, high RoB, or some concerns of bias. RoB assessment was performed independently by two authors (JJB and FMT), and discrepancies were resolved by discussion with the fourth author (AVH).

### 2.7. Statistical Analysis

Effects were described as mean differences (MD) for continuous outcomes and relative risks (RR) for dichotomous outcomes, with their confidence intervals (95% CIs). Inverse variance method and random effects model were used to assess the effects of TUN vs. CAF on primary and secondary outcomes. The between-study variance was estimated using the Paule–Mandel method. Heterogeneity of effects among RCTs was described with the *I*^2^ statistic, with the following degrees: 0%–30% (low), 30%–60% (moderate), and >60% (high). We performed subgroup analyses by type of graft (ADM vs. CTG) for primary and secondary outcomes. The metabin and metacont functions of the meta library of R 3.5.1 (https://www.r-project.org) were used for all analyses; *p* < 0.05 was considered statistically significant [15].

We also used the Grading of Recommendations, Assessment, Development and Evaluations (GRADE) methodology evaluate the quality of evidence (QoE) per outcome [16]. Five aspects were evaluated per outcome: RoB, indirectness, imprecision, inconsistency, and publication bias; the QoE was classified as high, moderate, low, and very low. QoE was described in summary of finginds (SoF) tables; GRADEpro GDT (https://gradepro.org/, McMaster University and Evidence Prime, Inc., 2020) was used to create SoF tables.

## 3. Results

### 3.1. Selection of Studies

**Figure 1:**
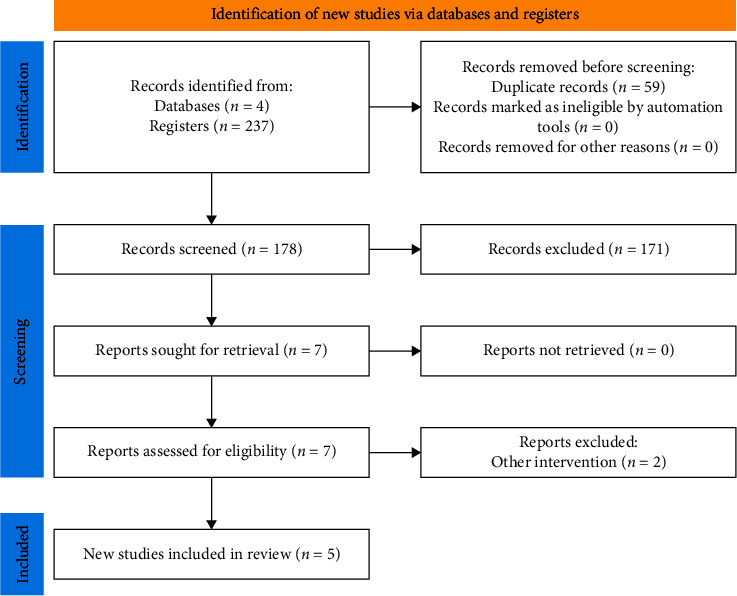
PRISMA flowchart of the study selection process.

A total of 237 abstracts were identified; 59 duplicated abstracts were excluded. Among the 178 selected abstracts, 171 manuscripts were excluded after title and abstract review. Seven full-text studies were assessed for eligibility and two were excluded due to assessing other interventions. Finally, five RCTs (*n* = 173) were included for qualitative and quantitative analyses (Figure 1) [16, 17, 19–21].

### 3.2. Characteristics of Included Trials

Studies were conducted in the United States [17, 21], Brazil [16, 20], and Turkey [19]. The age range was 18–56 years. All the studies followed patients up until 6 and 12 months after surgery (Table 1). The main Miller class described across trials was I or II buccal GR localized at upper incisors, canines, or premolars. One study compared TUN + CTG vs. CAF + CTG [20] and four studies compared TUN + ADM vs. CAF + ADM [16, 17, 19, 21].

### 3.3. Risk of Bias Assessment

Overall, three RCTs were at high RoB 2.0 [17, 19, 21]. Three RCTs were at high RoB in the randomization process [17, 19, 21], and one RCT was at high RoB in deviations from intended interventions [19]. The other RCTs showed some concerns of bias and low RoB in missing outcome data and selection of the reported result (Supplementary Figure S1).

### 3.4. Effect of TUN on Primary Outcomes

**Figure 2:**
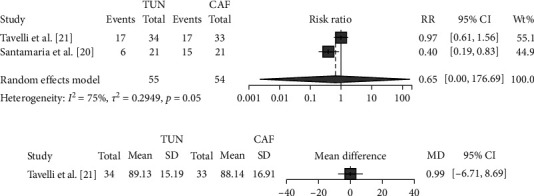
Effects of TUN vs. CAF on primary outcomes: (a) CRC; (b) RC.

In comparison with CAF, TUN did not significantly reduce CRC (RR 0.65; 95% CI 0.002–176.7; *p* = 0.51; *I*^2^ = 75%; Figure 2(a)) and did not increase RC (MD 0.99%; 95% CI −6.7 to 8.6; *p* = 0.80; Figure 2(b)).

### 3.5. Effects of TUN on Secondary Outcomes

**Figure 3:**
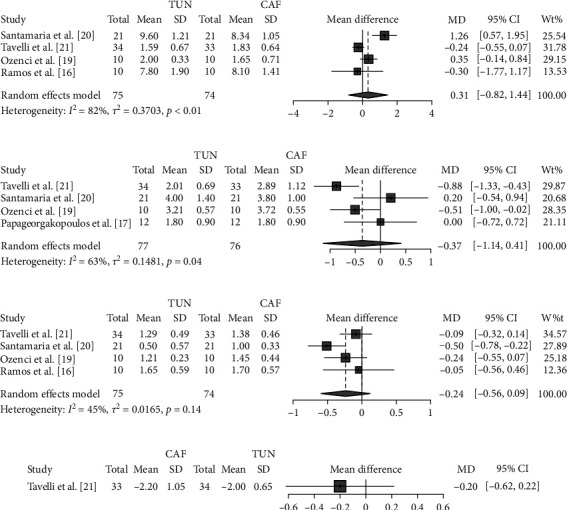
Effects of TUN vs. CAF on secondary outcomes: (a) CAL (mm); (b) KTW (mm); (c) PD (mm); (d) REC (mm).

In comparison with CAF, TUN did not significantly reduce CAL (MD 0.31 mm; 95% CI −0.8 to 1.4; *p* = 0.45; *I*^2^ = 82%; Figure 3(a)), KTW (MD −0.37 mm; 95% CI −1.14 to 0.41; *p* = 0.23; *I*^2^ = 63%; Figure 3(b)), PD (MD −0.24 mm; 95% CI −0.56 to 0.09; *p* = 0.10; *I*^2^ = 45% Figure 3(c)), and REC (MD −0.20 mm; 95% CI −0.62 to 0.22; *p* = 0.35; Figure 3(d)).

### 3.6. Subgroup Analyses

Subgroup analyses showed no significant differences in comparison to primary analyses for primary and secondary outcomes by type of graft (ADM or CTG) (Supplementary Figures S2–S7).

### 3.7. Quality of Evidence

QoE was very low for all primary and secondary outcomes (Supplementary Table S1). In CRC, RC, CAL, KTW, PD, REC, and the QoE was very low due to high RoB, inconsistency, and imprecision of effects.

## 4. Discussion

### 4.1. Main Findings

In our systematic review and meta-analysis, we found that TUN did not significantly increase CRC and did not significantly decrease RC, CAL, KTW, PD, and REC compared to CAF. There were no changes in effects when subgroups by type of graft were evaluated. QoE was very low for primary and secondary outcomes due to high RoB, inconsistency, and imprecision of effects.

### 4.2. What is Known in the Literature about the Research Question?

GR is the displacement of the GM apical to the CEJ [22–24]. Factors associated with this recession can be a thin gingival phenotype, excessive force when brushing teeth, cervical restorations, and orthodontic treatment [25]. Currently, there are several interventions for the treatment of GR [26]. The treatment of GR has become an important problem in periodontal surgery, since it is highly prevalent, especially in patients with risk factors [27–29]. CAF is a technique that can be performed alone or in combination with CTG [27]. Generally, CAF consists of making two oblique incisions, starting from the angle of the distal and mesial line of the affected tooth, directing them apically into the alveolar mucosa, and then the flap is displaced coronally [30–32]. Another option to treat GR is TUN that can be prepared in full or partial thickness [33]. In most cases with GR, gingival tissues are thin, therefore, a total thickness flap design is needed, which is the safest method to avoid breakage and tearing [34]. TUN and CAF have strengths and weaknesses. Advantages of CAF include better visibility and access in dissection, graft stabilization, and periosteal elevation [34]; meanwhile, TUN generates greater preservation of the gingival papillae and has faster healing and provides better blood nutrition to the graft that translates into more esthetic results than CAF. The main weaknesses of both techniques are requiring additional training and using of specialized surgical material [35, 36].

Both TUN and CAF have shown similar improvement in gingival esthetics and reduction in root exposure. For example, in a recent trial by Salhi et al. [37], they found that after 6 months, no difference was observed between CAF and TUN. It also known that soft tissue grafts play an important role in the reconstruction of the marginal gingiva and papillae. According to Chen and Zhang [38], there are currently novel techniques such as TUN that are more conservative in their performance, since they do not require extensive incisions and could mainly improve the RC in the GRs.

A recent systematic review and meta-analysis by Tavelli et al. [12] in patients with multiple or localized GR defects were published. The authors included 20 studies (11 RCTs and 9 case series; 1,181 recessions treated with TUN), with a follow-up period of 11 months, but only six RTCs were considered in the meta-analysis. The authors searched in three engines (PubMed, EMBASE, Cochrane Oral Health Group Trials Register). Their primary comparison was TUN vs. CAF comparison and included multiple types of graft. Also, Tavelli et al. [12] assessed RC and CRC as primary outcomes; secondary outcomes were KTW and root coverage esthetic score (RES). CAF and TUN obtained comparable results in terms of RC, CRC, and KTW when different types of graft material were evaluated. However, CAF showed better results to TUN when ADM was used. However, the evaluation periods among the studies evaluated by Tavelli et al. [12] were very heterogeneous as they presented a follow-up of 4, 6, and 12 months.

Tavelli et al. [12] found no statistically significant difference between TUN and CAF for RC, which was reported as rate. RC between the TUN and CAF groups was not different (MD 4.38 mm, 95% CI −9.06, 17.83; *p* = 0.52, *I*^2^ = 93%). However, when subgroup analyses were performed for those using ADM as graft, a statistically significant difference in RC was observed in favor of CAF (MD 17.99 mm, 95% CI 12.79, 23.19) with low heterogeneity between results (*I*^2^ = 0%). Also, according to Tavelli et al. [12], CRC was similar between arms (RR 0.74, 95% CI −0.66, 2.14, *p* = 0.3) with a high heterogeneity between articles (*I*^2^ = 82%). However, subgroup analyses by type of graft (CTG or ADM) revealed significant effects in favor of CAF. Low heterogeneity was observed for subgroup analyses in the CTG and ADM groups. Finally, they found no significant difference in changes of KTW when comparing TUN and CAF (MD −0.09 mm, 95% CI −0.50, 0.32; *p* = 0.6). However, when subgroup analyses were performed with ADM graft material, there was a significant difference in KTW in favor of CAF (MD 0.36 mm, 95% CI 0.20, 0.52; *p* < 0.001) with low heterogeneity [12].

### 4.3. What Our Study Adds to the Literature

In our systematic review, we only focused on the evaluation of RCTs. We included single and multiple recession types, and we excluded those RCT studies that did not evaluate TUN vs. CAF. Furthermore, we only included studies that evaluated TUN vs. CAF using ADM or CTG as a complementary graft to these techniques for the treatment of GRs, evaluating the same primary outcomes of Tavelli et al. [12]. However, our set of secondary outcomes was different because we evaluated other periodontally important clinical outcomes, such as CRC, KTW, CAL, PD, and REC, that allow a better measurement and evaluation of gingival lesions in the periodontal specialty. On the other hand, in our study, some effects were different from those described by Tavelli et al. [12]; this discrepancy is probably attributed to the fact that in our study we did not differentiate GR by location (upper or lower jaw). Furthermore, we did not find significant effects of TUN vs. CAF on the primary outcomes CRC and RC nor on the secondary outcomes, several of which were also not evaluated in the study by Tavelli et al. [12]. Finally, unlike the meta-analysis [12], our study performed an assessment of the QoE and found it to be very low for most primary and secondary outcomes (CRC, CAL, KTW, PD, and REC).

Also, we created better search strategies with full sets of MeSH terms and Emtree terms of Embase available in five databases, and we evaluated updated studies until September 2021. Also, we used the Cochrane Collaboration RoB 2.0 tool to assess RoB, which is a more up-to-date version than the older 2011 RoB tool. In addition, we performed subgroup analyses by graft type and found no differences with overall analyses. Finally, we used GRADE methodology to assess QoE of all outcomes across RCTs.

### 4.4. Limitations

There are some limitations in our study. First, there were a few RCTs comparing TUN vs. CAF with ADM injection and/or CTG; the total number of evaluated individuals was small. Second, there were differences in follow-up times across RCTs; however, all outcomes of interest were reported at 6 months. Third, the RCTs included in our study the same techniques of TUN or CAF, but there were some characteristics of their application, which have been detailed in Table 1. Fourth, the ADM and CTG grafts were the same in all included studies but had some individual specifications [17, 23]. Finally, the QoE per GRADE evaluation was very low for most outcomes, due to high heterogeneity among effects, imprecision of effects, and a high RoB in most of RCTs [39].

## 5. Conclusion

TUN had similar primary and secondary outcomes compared to CAF. Subgroup analyses by type of graft did not affect the main conclusions. However, the QoE was very low for most of the outcomes. More RCTs with better design are needed to better characterize the effects of TUN vs. CAF in the treatment of multiple GR defects.

## Figures and Tables

**Table 1:** Characteristics of included randomized controlled trials.

Author, year	Country	Length of follow-up	Sample size	Age (SD)	Miller class	Control	Intervention	Evaluated outcomes	Finding
Santamaria et al., 2017 [20]	Brazil	Baseline, 3 months, 6 months	42	40.2 ± 9.6 years	Miller's class I or II gingival recession in maxillary canine and premolar	TUN + CTG	CAF + CTG	CRC, REC-reduction, GRD, mRC, RES, KTT, KTW, PD, and VAS	CAF + CTG and TUN + CTG could reduce GR and improve aesthetics in defects
Tavelli et al., 2019 [21]	United States	From 6 months to 12 months	67	18 years old	Miller class I or II and cairo (RT1) maxillary incisors, canines, or premolars	TUN + ADM	CAF + ADM	REC, PD, CAL, CRC, mRC, KTW, and GT	There was a significant gain in gingival margin when GR was treated with ADM
Ozenci et al., 2015 [19]	Turkey	Baseline and 12 months	20	30.7 ± 5.9 years	Miller's class I in maxillary or mandibular canines, premolars, or incisors	TUN + ADM	CAF + ADM	mRC, CRC, PI, GI, BoP, PD, CAL, RH, RW, GT, and KTH	Better clinical results were obtained with the combination of CAF + ADM although both techniques were effective in the treatment of GR
Ramos et al., 2022 [16]	Brazil	Baseline and 6 months	20	18–59 years	Miller class III	CAF + ADM	TUN + ADM	PD, rCAL, GR, KT, TKT, and GRA	Both CAF + ADM and TUN + ADM were effective in root coverage of GR
Papageorgakopoulos et al., 2008 [17]	United States	After surger, patients were evaluated for 8 weeks, and then monthly until the end of the study period	24	40 ± 13 years	Miller class I or II maxillary and mandibular canines and premolars	TUN + ADM	CAF + ADM	KTT, PD, GT, GR, and CA	Both CAF + CTG and TUN + CTG could reduce GR

CAF, coronally advanced flap; TUN, tunnel technique; ADM, acellular dermal matrix; GR, gingival recession; CRC, complete root coverage; CAL, clinical attachment level; KTW, keratinized tissue width; PD, probing depth; REC, recession coverage; mRC, mean root coverage; RC, root coverage; GT, gingival thickness; CTG, connective tissue graft; KTT, keratinized tissue thickness; PPD, pocket probing depth; WKT, width of keratinized tissue; STT, soft tissue thickness; HKT, height of keratinized tissue: GI, gingival index; CEJ, connective junctional epithelium; VAS, visual analog scale; RES, root esthetic score; PI, plaque index; GI, gingival index; BoP, bleeding on probing; RH, recession height; RW, recession width; CA, creeping attachment; TKT, thickness of keratinized tissue; GRA, gingival recession area; rCAL, relative clinical attachment level; KT, width of keratinized tissue.

## Data Availability

The data for supporting this review were taken from previously studies. Data are available upon request to the corresponding author.

## Supplementary Materials

Supplementary Materials Figure S1: risk of bias assessment of included trials. Figure S2: subgroup analyses by type of graft on complete RC. Figure S3: subgroup analyses by type of graft on mean root coverage. Figure S4: subgroup analyses by type of graft on clinical attachment level. Figure S5: subgroup analyses by type of graft on keratinized tissue width. Figure S6: subgroup analyses by type of graft on probing depth. Figure S7: subgroup analyses by type of graft on recession coverage (REC). Table S1: GRADE summary of findings table.

## References

[1] Chrysanthakopoulos N. A. (2014). Gingival recession: prevalence and risk indicators among young Greek adults. *Journal of Clinical and Experimental Dentistry*.

[2] Żurek J., Dominiak M., Tomaszek K., Botzenhart U., Gedrange T., Bednarz W. (2016). Multiple gingival recession coverage with an allogeneic biostatic fascia lata graft using the tunnel technique—a histological assessment. *Annals of Anatomy - Anatomischer Anzeiger*.

[3] Chrysanthakopoulos N. A. (2011). Aetiology and severity of gingival recession in an adult population sample in Greece. *Dental Research Journal (Isfahan)*.

[4] Mythri S., Arunkumar S. M., Hegde S., Rajesh S. K., Munaz M., Ashwin D. (2015). Etiology and occurrence of gingival recession—an epidemiological study. *Journal of Indian Society of Periodontology*.

[5] Heasman P. A., Holliday R., Bryant A., Preshaw P. M. (2015). Evidence for the occurrence of gingival recession and non-carious cervical lesions as a consequence of traumatic toothbrushing. *Journal of Clinical Periodontology*.

[6] Kareem N., Mahendra J., Kumar K. A. (2018). Triangular coronally advanced flap: conventional versus microsurgery. *Journal of Indian Society of Periodontology*.

[7] de Sanctis M., Zucchelli G. (2007). Coronally advanced flap: a modified surgical approach for isolated recession-type defects: three-year results. *Journal of Clinical Periodontology*.

[8] Fahmy R. A., Taalab M. R. (2018). Modified tunnel technique for management of gingival recession in esthetic zone using acellular dermal matrix versus connective tissue graft. *Future Dental Journal*.

[9] Dani S., Dhage A., Gundannavar G. (2014). The pouch and tunnel technique for management of multiple gingival recession defects. *Journal of Indian Society of Periodontology*.

[10] Pazmiño V. F. C., Rodas M. A. R., Cáceres C. D. B. (2017). Clinical comparison of the subepithelial connective tissue versus platelet-rich fibrin for the multiple gingival recession coverage on anterior teeth using the tunneling technique. *Case Reports in Dentistry*.

[11] Barakat H., Dayoub S., Alarkan R. (2018). A porcine collagen matrix (mucograft®) vs connective tissue graft in the treatment of multiple gingival recessions: a comparative clinical study. *World Journal of Dentistry*.

[12] Tavelli L., Barootchi S., Nguyen T. V. N., Tattan M., Ravidà A., Wang H.-L. (2018). Efficacy of tunnel technique in the treatment of localized and multiple gingival recessions: a systematic review and meta-analysis. *Journal of Periodontology*.

[13] Page M. J., McKenzie J. E., Bossuyt P. M. (2021). The PRISMA 2020 statement: an updated guideline for reporting systematic reviews. *BMJ*.

[14] Sterne J. A. C., Savović J., Page M. J. (2019). RoB 2: a revised tool for assessing risk of bias in randomised trials. *BMJ*.

[15] Mathur M. B., VanderWeele T. J. (2020). Sensitivity analysis for unmeasured confounding in meta-analyses. *Journal of the American Statistical Association*.

[16] Ramos U. D., Bastos G. F., Costa C. A., de Souza S. L. S., Taba M., Novaes A. B. (2022). Root coverage with tunneling technique or modified advanced flap associated with acellular dermal matrix: results from 6 months randomized clinical trial. *Clinical Oral Investigations*.

[17] Papageorgakopoulos G., Greenwell H., Hill M., Vidal R., Scheetz J. P. (2008). Root coverage using acellular dermal matrix and comparing a coronally positioned tunnel to a coronally positioned flap approach. *Journal of Periodontology*.

[18] Zuhr O., Rebele S. F., Vach K., Petsos H., Hürzeler M. B., Research Group for Oral Soft Tissue Biology & Wound Healing (2020). Tunnel technique with connective tissue graft versus coronally advanced flap with enamel matrix derivate for root coverage: 2-year results of an RCT using 3D digital measuring for volumetric comparison of gingival dimensions. *Journal of Clinical Periodontology*.

[19] Ozenci I., Ipci S. D., Cakar G., Yilmaz S. (2015). Tunnel technique *versus* coronally advanced flap with acellular dermal matrix graft in the treatment of multiple gingival recessions. *Journal of Clinical Periodontology*.

[20] Santamaria M. P., da Silva Neves F. L., Silveira C. A. (2017). Connective tissue graft and tunnel or trapezoidal flap for the treatment of single maxillary gingival recessions: a randomized clinical trial. *Journal of Clinical Periodontology*.

[21] Tavelli L., Barootchi S., Di Gianfilippo R. (2019). Acellular dermal matrix and coronally advanced flap or tunnel technique in the treatment of multiple adjacent gingival recessions. A 12-year follow-up from a randomized clinical trial. *Journal of Clinical Periodontology*.

[22] Bherwani C., Kulloli A., Kathariya R. (2014). Zucchelli’s technique or tunnel technique with subepithelial connective tissue graft for treatment of multiple gingival recessions. *Journal of the International Academy of Periodontology*.

[23] Azaripour A., Kissinger M., Farina V. S. L. (2016). Root coverage with connective tissue graft associated with coronally advanced flap or tunnel technique: a randomized, double-blind, mono-centre clinical trial. *Journal of Clinical Periodontology*.

[24] Xue F., Zhang R., Cai Y., Zhang Y., Kang N., Luan Q. (2021). Three-dimensional quantitative measurement of buccal augmented tissue with modified coronally advanced tunnel technique and de-epithelialized gingival graft: a prospective case series. *BMC Oral Health*.

[25] Vincent-Bugnas S., Borie G., Charbit Y. (2018). Treatment of multiple maxillary adjacent class I and II gingival recessions with modified coronally advanced tunnel and a new xenogeneic acellular dermal matrix. *Journal of Esthetic and Restorative Dentistry*.

[26] Rimbert M., Barré R. (2021). New surgical approach for mandibular anterior root coverage by modified tunnel technique with simultaneous frenuloplasty: technical description and 5-year recall case report. *Clinical Advances in Periodontics*.

[27] Khanna D., George J. P., Babrawala I. S., Divakaran R., Bhardwaj S., Chakraborty P. (2017). Treatment of multiple gingival recessions using a minimally invasive coronally advanced tunnel: a randomized controlled clinical trial. *Journal of the International Academy of Periodontology*.

[28] Garzon H. S., Alfonso C., Vega F. J. (2021). Treatment of miller I mandibular gingival recessions using PRF vs. connective graft. *International Journal of Dentistry*.

[29] Imber J.-C., Kasaj A. (2021). Treatment of gingival recession: when and how?. *International Dental Journal*.

[30] Aroca S., Barbieri A., Clementini M., Renouard F., de Sanctis M. (2018). Treatment of class III multiple gingival recessions: prognostic factors for achieving a complete root coverage. *Journal of Clinical Periodontology*.

[31] Cairo F. (2017). Periodontal plastic surgery of gingival recessions at single and multiple teeth. *Periodontology 2000*.

[32] Maluta R., Monteiro M. F., Peruzzo D. C., Joly J. C. (2021). Root coverage of multiple gingival recessions treated with coronally advanced flap associated with xenogeneic acellular dermal matrix or connective tissue graft: a 6-month split-mouth controlled and randomized clinical trial. *Clinical Oral Investigations*.

[33] Górski B., Górska R., Wysokińska-Miszczuk J., Kaczyński T. (2020). Tunnel technique with enamel matrix derivative in addition to subepithelial connective tissue graft compared with connective tissue graft alone for the treatment of multiple gingival recessions: a randomized clinical trial. *Clinical Oral Investigations*.

[34] Aroca S., Di Domenico G. L., Darnaud C., de Sanctis M. (2021). Modified coronally advanced tunnel technique with site-specific application of connective tissue graft for the treatment of multiple adjacent maxillary gingival recessions: a case series. *The International Journal of Periodontics & Restorative Dentistry*.

[35] Dandu S. R., Murthy R. V. (2016). Multiple gingival recession defects treated with coronally advanced flap and either the VISTA technique enhanced with GEM 21S or periosteal pedicle graft: a 9-month clinical study. *The International Journal of Periodontics & Restorative Dentistry*.

[36] Pini Prato G. P., Magnani C., Chambrone L. (2018). Long-term evaluation (20 years) of the outcomes of coronally advanced flap in the treatment of single recession-type defects. *Journal of Periodontology*.

[37] Salhi L., Lecloux G., Seidel L., Rompen E., Lambert F. (2014). Coronally advanced flap versus the pouch technique combined with a connective tissuegraft to treat Miller’s class I gingival recession: a randomized controlled trial. *Journal of Clinical Periodontology*.

[38] Chen T. L., Zhang X. H. (2017). The role of soft tissue transplantation in the reconstruction of gingival papilla. *Zhonghua Kou Qiang Yi Xue Za Zhi*.

[39] Barboza J. J., Huamán M. R., Melgar B., Diaz-Arocutipa C., Valenzuela-Rodriguez G., Hernandez A. V. (2022). Efficacy of liraglutide in non-diabetic obese adults: a systematic review and meta-analysis of randomized controlled trials. *Journal of Clinical Medicine*.

