# Supplementary material for: Efficacy of Tunnel Technique (TUN) versus Coronally Advanced Flap (CAF) in the Management of Multiple Gingival Recession Defects: A Meta-Analysis
Source: Int J Dent. 2023 Apr 6;2023:8671484. doi: 10.1155/2023/8671484 (PMC10101741; doi:10.1155/2023/8671484)
Supplement: Supplementary Materials — Figure S1: risk of bias assessment of included trials. Figure S2: subgroup analyses by type of graft on complete RC. Figure S3: subgroup analyses by type of graft on mean root coverage. Figure S4: subgroup analyses by type of graft on clinical attachment level. Figure S5: subgroup analyses by type of graft on keratinized tissue width. Figure S6: subgroup analyses by type of graft on probing depth. Figure S7: subgroup analyses by type of graft on recession coverage (REC). Table S1: GRADE summary of findings table. [file 8671484.f1.docx]

**Efficacy of tunnel technique (TUN) vs. coronally advanced flap (CAF) in the management of gingival recession: a meta-analysis**

Frank Mayta-Tovalino, Joshuan J. Barboza, Vinay Pasupuleti, Adrian V. Hernandez.

**Supplementary contents**

**Search strategies for Pubmed, Scopus and Web of Science**

- **Supplemental Figure S1:** Risk of bias assessment of included trials
- **Supplemental Figure S2:** Subgroup analyses by type of graft on complete root coverage
- **Supplemental Figure S3:** Subgroup analyses by type of graft on mean root coverage
- **Supplemental Figure S4:** Subgroup analyses by type of graft on clinical attachment level
- **Supplemental Figure S5:** Subgroup analyses by type of graft on keratinized tissue width
- **Supplemental Figure S6:** Subgroup analyses by type of graft on probing depth
- **Supplemental Figure S6:** Subgroup analyses by type of graft on recession coverage
- **Supplemental Figure S7:** Subgroup analyses by type of graft on recession coverage (REC)
- **Supplemental Table T1:** GRADE summary of findings table
- **Search strategy for Pubmed, Scopus and Web of Science**

**PUBMED**

1. (“Gingival recession” OR “Gingival Recessions” OR “Recession, Gingival” OR “Recessions, Gingival” OR “Gingival Atrophy” OR “Gingival Atrophies” OR “Atrophy of Gingiva” OR “Gingiva Atrophies” OR “Gingiva Atrophy” OR “Gingival diseases” OR “Gingival inflammation”)
2. (“Acellular dermal matrix” OR “Acellular dermis” OR “Acellular human dermis” OR “Allopatch” OR “Epiflex” OR “MatriStem Burn Matrix” OR “MatriStem MicroMatrix” OR “MatriStem Multilayer Wound Matrix” OR “MatriStem UBM” OR “MatriStem Wound Matrix” OR “PriMatrix” OR “SureDerm” OR “SurgiMend” OR “Xe-Derma”)
3. (“Connective Tissue Graft” OR “Connective tissue cell” OR “subepithelial connective tissue graft” OR “connective tissue grafts” OR “connective tissue graft technique”)
4. (“Coronally advanced flap” OR “CAF”)
5. (“Tunnel technique” OR “TUN”)
6. #1 AND #2 OR #3 AND #4 AND # 5

**SCOPUS**

1. ALL (“Gingival recession” OR “Gingival Recessions” OR “Recession, Gingival” OR “Recessions, Gingival” OR “Gingival Atrophy” OR “Gingival Atrophies” OR “Atrophy of Gingiva” OR “Gingiva Atrophies” OR “Gingiva Atrophy” OR “Gingival diseases” OR “Gingival inflammation”)
2. ALL (“Acellular dermal matrix” OR “Acellular dermis” OR “Acellular human dermis” OR “Allopatch” OR “Epiflex” OR “MatriStem Burn Matrix” OR “MatriStem MicroMatrix” OR “MatriStem Multilayer Wound Matrix” OR “MatriStem UBM” OR “MatriStem Wound Matrix” OR “PriMatrix” OR “SureDerm” OR “SurgiMend” OR “Xe-Derma”)
3. ALL (“Connective Tissue Graft” OR “Connective tissue cell” OR “subepithelial connective tissue graft” OR “Connective tissue grafts” OR “connective tissue graft technique”)
4. (“Coronally advanced flap” OR “CAF”)
5. (“Tunnel technique” OR “TUN”)
6. #1 AND #2 OR #3 AND #4 AND # 5

**WOS**

1. ALL (“Gingival recession” OR “Gingival Recessions” OR “Recession, Gingival” OR “Recessions, Gingival” OR “Gingival Atrophy” OR “Gingival Atrophies” OR “Atrophy of Gingiva” OR “Gingiva Atrophies” OR “Gingiva Atrophy” OR “Gingival diseases” OR “Gingival inflammation”)

2. ALL (“Acellular dermal matrix” OR “Acellular dermis” OR “Acellular human dermis” OR “Allopatch” OR “Epiflex” OR “MatriStem Burn Matrix” OR “MatriStem MicroMatrix” OR “MatriStem Multilayer Wound Matrix” OR “MatriStem UBM” OR “MatriStem Wound Matrix” OR “PriMatrix” OR “SureDerm” OR “SurgiMend” OR “Xe-Derma”)

3. ALL (“Connective Tissue Graft” OR “Connective tissue cell” OR “subepithelial connective tissue graft” OR “Connective tissue grafts” OR “connective tissue graft technique”)

4. (“Coronally advanced flap” OR “CAF”)

5. (“Tunnel technique” OR “TUN”)

6. #1 AND #2 AND #3 AND #4 AND # 5

**Supplemental Figure S1:** Risk of bias assessment of included trials


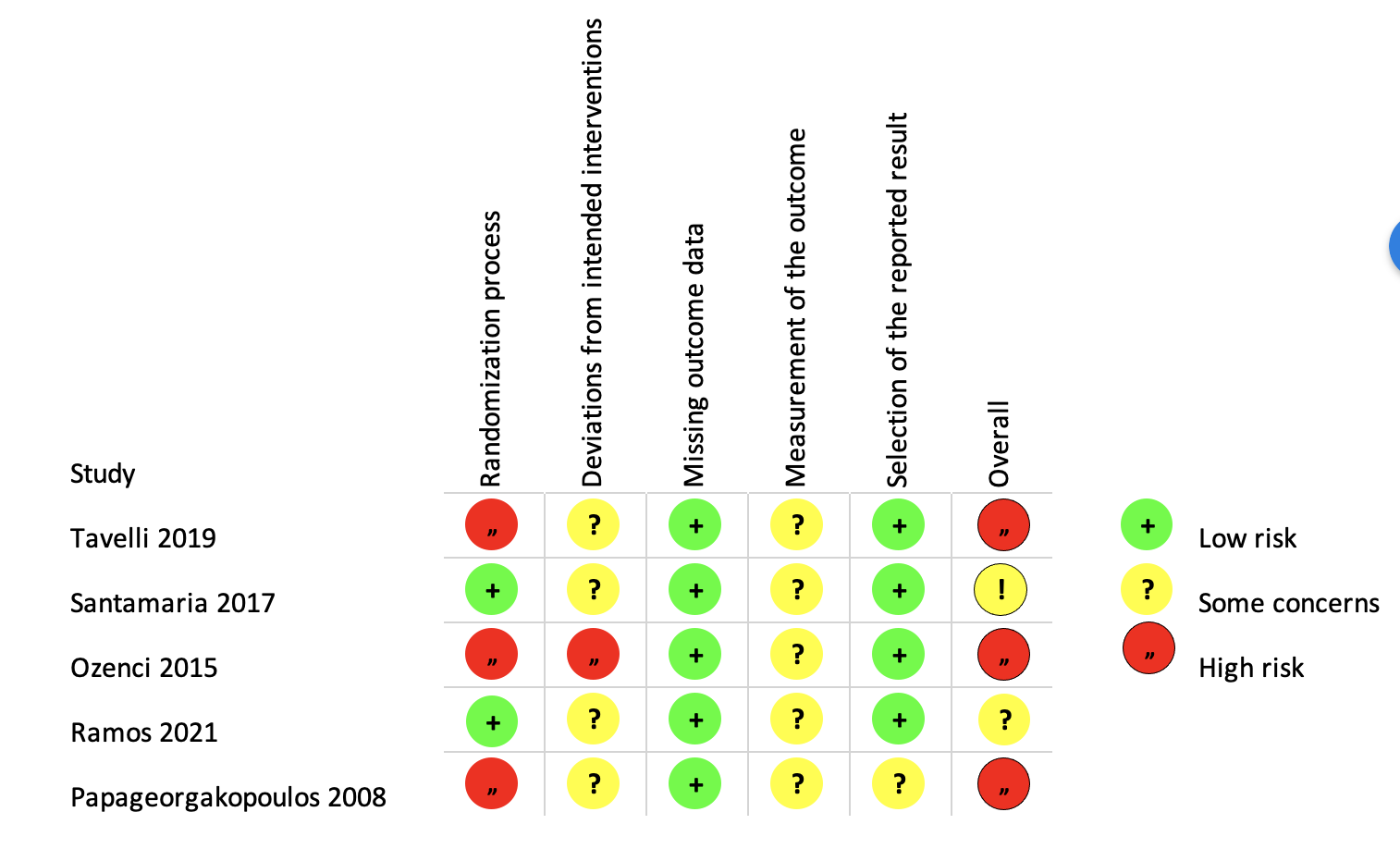


- **Supplemental Figure S2:** Subgroup analyses by type of graft on complete root coverage (CRC)

**
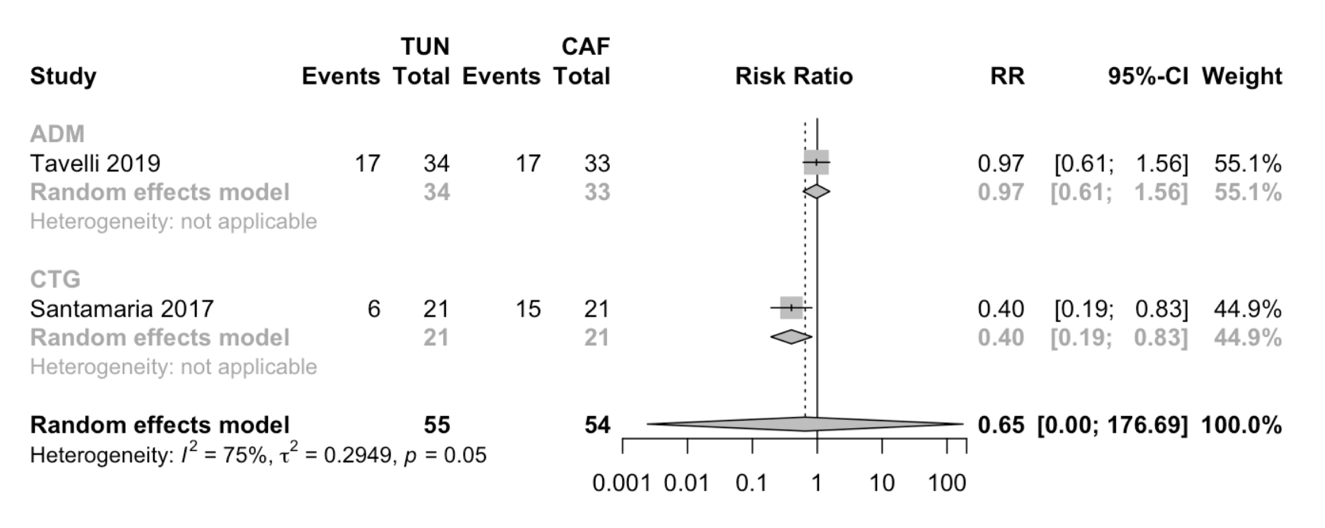
**

- **Supplemental Figure S3:** Subgroup analyses by type of graft on mean root coverage (RC)


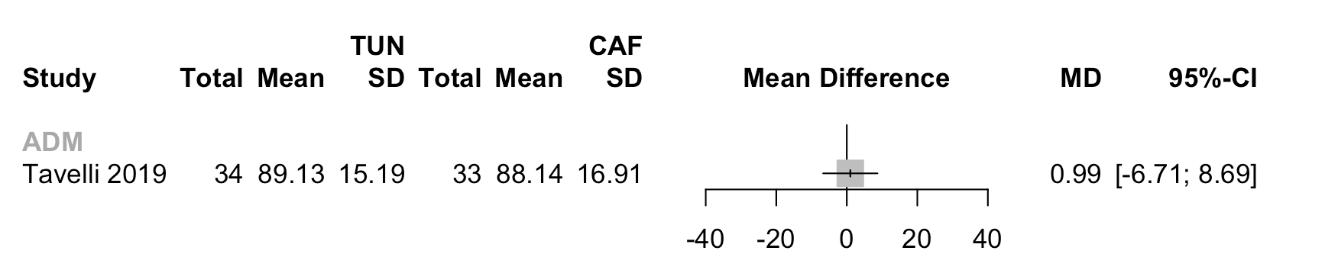


- **Supplemental Figure S4:** Subgroup analyses by type of graft on clinical attachment level (CAL)


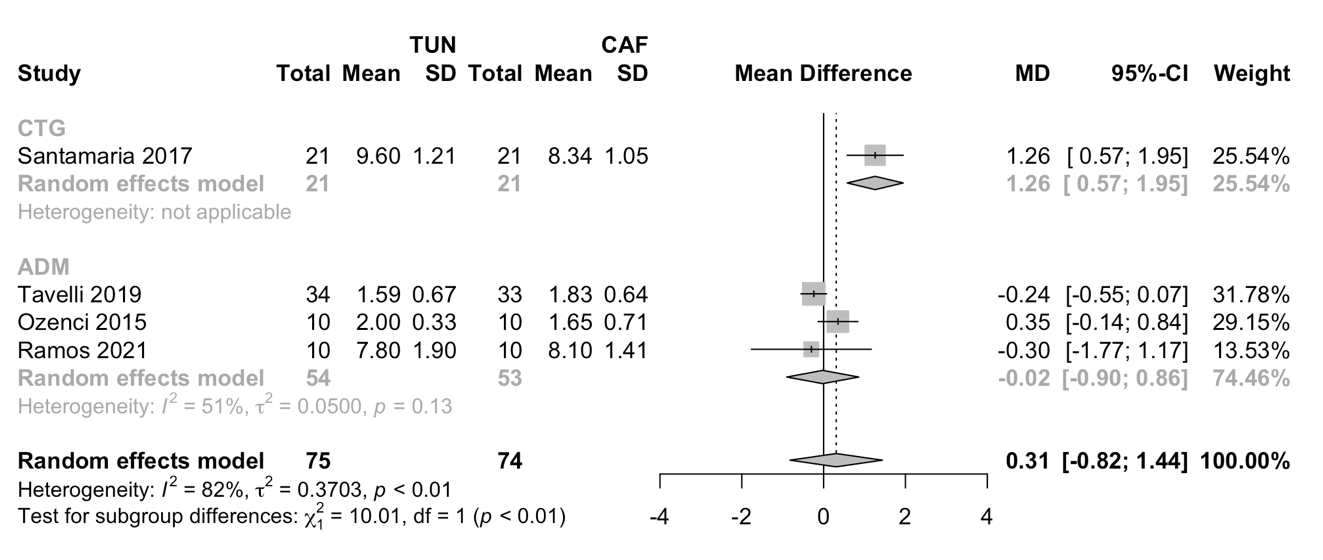


- **Supplemental Figure S5:** Subgroup analyses by type of graft on keratinized tissue width (KTW)


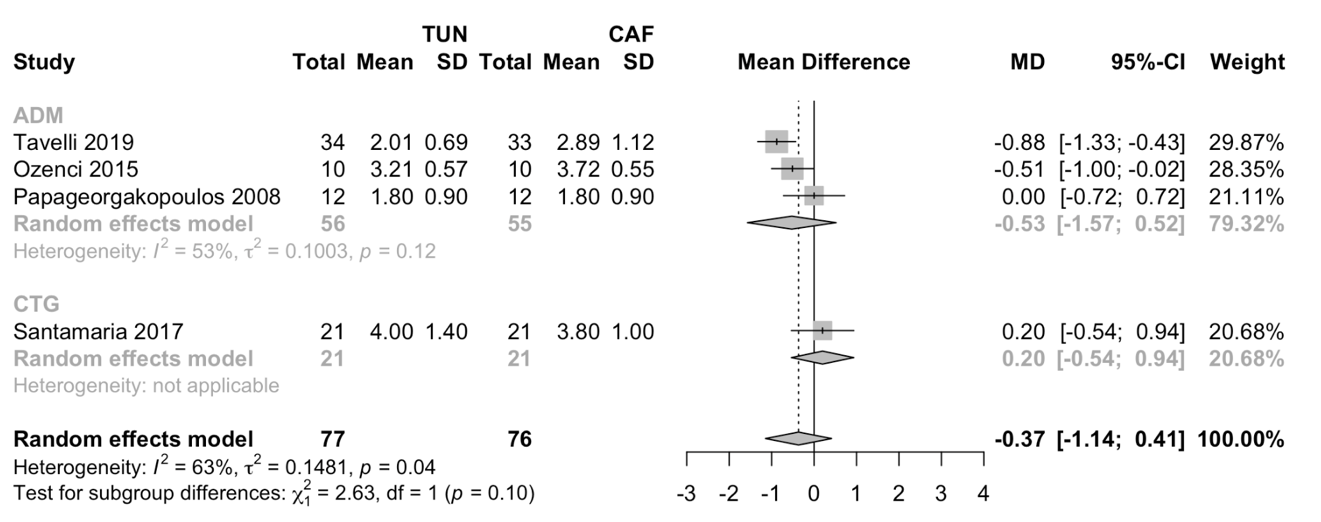


- **Supplemental Figure S6:** Subgroup analyses by type of graft on probing depth (PD)


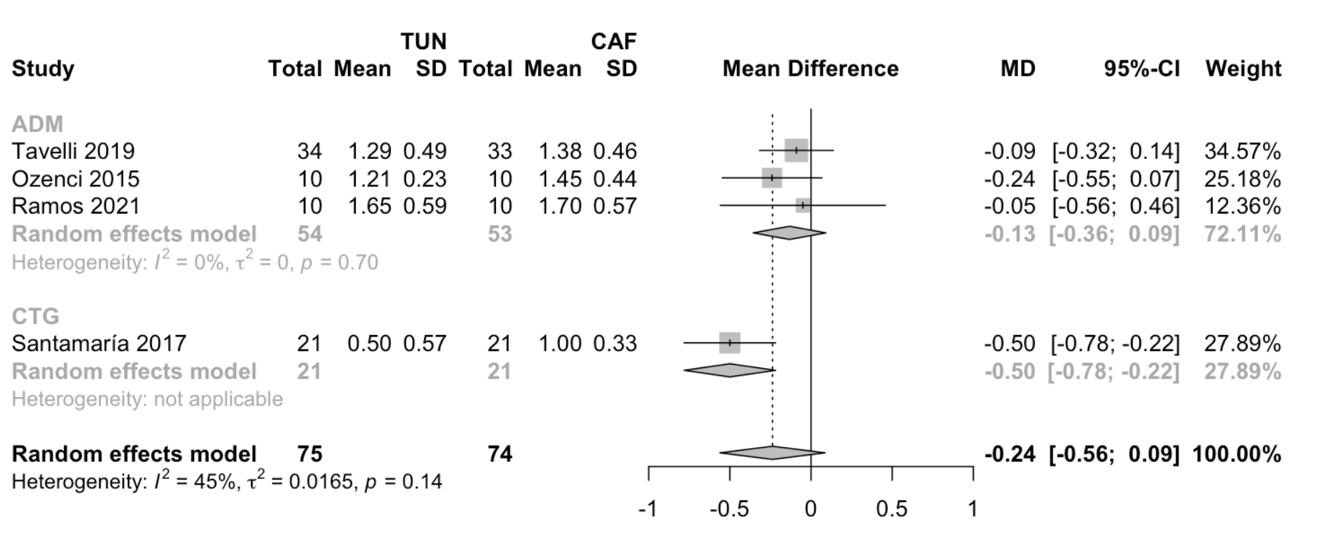


**Supplemental Figure S7:** Subgroup analyses by type of graft on recession coverage (REC)


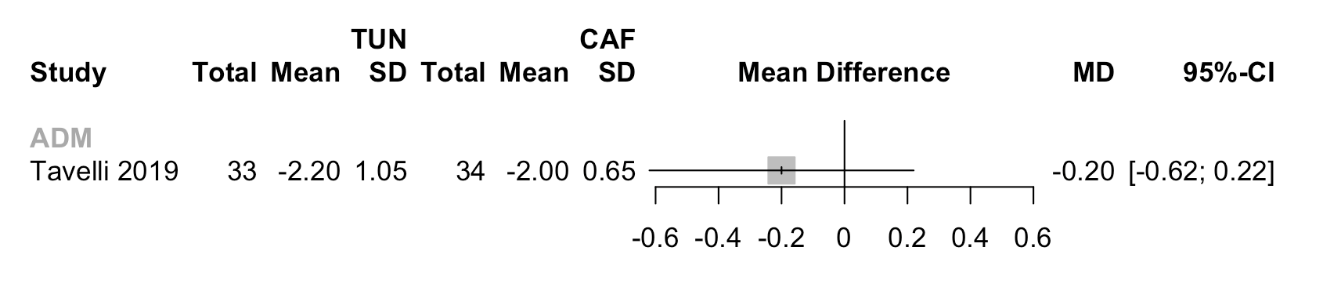


**Supplemental table T1: GRADE Summary of findings table of the effect of TUN vs. CAF on outcomes for the treatment of multiple adjacent gingival recession**

| **Certainty assessment** | | | | | | | **№ of patients** | | | **Effect** | | | **Certainty** |
| --- | --- | --- | --- | --- | --- | --- | --- | --- | --- | --- | --- | --- | --- |
| **№ of studies** | **Study design** | **Risk of bias** | **Inconsistency** | **Indirectness** | **Imprecision** | **Other considerations** | | **CAF** | **TUN** | | **Relative (95% CI)** | **Absolute (95% CI)** |  |
| 2 | randomised trials | very serious ^a^ | very serious ^b^ | not serious | very serious ^c^ | none | | 51/55 (92.7%) | 32/54 (59.3%) | | **RR 0.65** (0.00 to 176.60) | **207 fewer per 1,000** (from -- to 1,000 more) | ⨁◯◯◯ Very low |
| 2 | randomised trials | very serious ^a^ | serious ^d^ | not serious | very serious ^c^ | none | | 54 | 55 | | - | MD **0.47 mm lower** (4.52 lower to 3.58 higher) | ⨁◯◯◯ Very low |
| 2 | randomised trials | very serious ^a^ | very serious ^e^ | not serious | very serious ^c^ | none | | 54 | 55 | | - | MD **0.03 mm higher** (4.48 lower to 4.41 higher) | ⨁◯◯◯ Very low |
| 3 | randomised trials | very serious ^a^ | very serious ^f^ | not serious | very serious ^c^ | none | | 78 | 78 | | - | MD **0.1 mm lower** (1.09 lower to 0.89 higher) | ⨁◯◯◯ Very low |
| 1 | randomised trials | very serious ^a^ | very serious ^g^ | not serious | very serious ^c^ | none | | 33 | 34 | | - | MD **0.2 mm lower** (0.62 higher to 0.22 higher) | ⨁◯◯◯ Very low |
| 1 | randomised trials | very serious ^a^ | serious ^g^ | serious | serious ^c^ | none | | 34 | 33 | | - | MD **0.99 % higher** (6.7 lower to 8.6 higher) | ⨁◯◯◯ Very low |

**Explanations**

a. Downgraded one level by a high risk of bias in at least one of the studies for this outcome.

b. High heterogeneity. I2=81%

c. Downgraded two level due to serious imprecision because of wide confidence intervals in the studies and the 95% confidence interval includes both benefits and harms

d. High heterogeneity. I2=53%

e. High heterogeneity. I2=79%

f. High heterogeneity. I2=92%

g. Only one study
